# Acid-Adapted Polyphenol Oxidases from Agricultural Wastes: Extraction, Characterization, and Application in Plant Protein Crosslinking

**DOI:** 10.3390/foods14193312

**Published:** 2025-09-24

**Authors:** Trang Tran, Zhe Xu, John Coupland, Yi Zhang

**Affiliations:** 1 Department of Food Science, The Pennsylvania State University, University Park, PA 16802, USA; ttt5333@psu.edu (T.T.); zxx5155@psu.edu (Z.X.); jnc3@psu.edu (J.C.); 2 Huck Institutes of the Life Sciences, The Pennsylvania State University, University Park, PA 16802, USA

**Keywords:** acid-adapted PPOs, pear pomace, avocado seed, apple pomace, by-product valorization, plant protein crosslinking

## Abstract

Polyphenol oxidases (PPOs) are enzymes that oxidize mono- and diphenolic compounds to o-quinones, facilitating pigment formation and protein crosslinking in food systems, thereby improving their techno-functional properties. However, most PPOs function optimally near neutral pH, limiting their application in acidic food products. This study aimed to extract acid-adapted PPOs from various fruit by-products, including Hass avocado seeds (pH 5.9), Anjou pears (pH 4.0), Bartlett pears (pH 4.0), Red Delicious apples (pH 4.0), and McIntosh apples (pH 3.3), and characterize PPO properties and its substrate specificity using colorimetric assay. SDS-PAGE was used to assess PPOs’ molecular weight and PPOs’ capacity for plant protein crosslinking. The results showed that PPOs from Anjou and Bartlett pear pomace exhibited the most robust acid-adapted activity, with effective catalytic performance in the pH ranges of 4.0–5.0 and 5.0–8.0, respectively, and an optimal temperature of 20 °C. SDS-PAGE analysis revealed bands at ~44 kDa and ~25.6 kDa, consistent with previously found pear PPO isoforms. Both pear pomace PPO oxidized L-DOPA and EGCG efficiently, but showed minimal activity toward L-tyrosine, gallic acid, caffeic acid, tannic acid, and ferulic acid. In the presence of EGCG, both pear pomace PPOs are capable of crosslinking plant proteins at pH 4.0. These findings provide the first evidence that agricultural by-products are a promising but underutilized source of acid-adapted PPO for modifying soy protein hydrolysates.

## 1. Introduction

Polyphenol oxidase (PPO) is a common name for the copper-containing enzyme family which includes laccase (EC 1.10.3.2), tyrosinase (EC 1.14.18.1), and catechol oxidase (EC 1.10.3.1) [1]. These enzymes catalyze one or two primary reactions: the hydroxylation of monophenols to *o*-diphenols (monophenolase activity) and the oxidation of *o*-diphenols or *p*-diphenols to their corresponding quinones (diphenolase activity). The resulting quinones are reactive electrophiles capable of undergoing polymerization to form brownish pigments, an effect often considered undesirable in fruits and vegetables [2]. However, these quinones also react with amino acid side chains (e.g., lysyl, tyrosyl, or cysteinyl residues) to form covalent bonds within or between proteins. This crosslinking can alter the network structure of foods, thereby improving water retention, mechanical strength, and gel stability [3]. However, a major limitation for PPO in such applications, especially commercial tyrosinase from mushroom, is that they exhibit optimal activity at neutral or slightly basic pH [4], making them poorly suited for acidic food environments. Although certain fruits, such as cranberry, cherry, banana, plum, avocado, pear, and apple, naturally contain PPOs that retain some activity at a lower pH [2,5], most studies have focused on inhibiting their activity in fresh products rather than leveraging their functionality in food modification.

Apples, pears, and avocados are known for their enzymatic browning due to PPO activity. And PPO activity (both monophenolase and diphenolase activities) has been reported in apple pulp (*Malus domestica*) [6,7,8,9], pear pulp (var. *Blanquilla, Pyrus communis* L and *Pyrus pyrifolia*) [10,11,12], and avocado pulp (*Persea americana*) [6,13,14]. These fruits are also among the most widely consumed acidic fruits in the United States. Approximately 52% of apples are pressed into juice [15], around 40% of pears are juiced or used in processed products (e.g., canned) [16], and avocados are increasingly peeled and crushed before being frozen or treated with high pressure [17]. These processes generate large amounts of by-products, such as apple pomace, pear pomace, and avocado seeds, that are often underutilized [18]. About 4 million tons of apple pomace [19] and 613 thousand tons of avocado seeds (from the 4.71 million tons of avocado grown) are produced annually [20]. While commercially pure PPOs are costly and have optimal activity at neutral pH, restricting their large-scale use, crude PPO extracts from acidic agricultural by-products could offer a low-cost source of acid-adapted PPOs to be applied in acidic plant protein hydrolysates.

Soy protein is the most abundant and widely used plant protein, valued for its balanced essential amino acid composition and functional properties in food systems, including emulsification, gelation, and rheology [21,22]. Enzymatic hydrolysates of soy protein are generally more soluble, particularly at the soy protein isoelectric point (pH 4.6), exhibit enhanced emulsifying and foaming properties, and are used as functional food ingredients, flavor, and nutritious enhancers [23]. Previous studies have demonstrated that tyrosinase can catalyze the crosslinking of various soy protein forms to enhance emulsion stability, structural properties, and functionality; however, these reactions have all been conducted under neutral or alkaline pH conditions [22,24]. Although soy protein-based ingredients have been used in acidic plant-based food products, such as yogurts, beverages, and emulsified products like mayonnaise [25,26,27], no prior studies have explored the crosslinking of soy proteins or their hydrolysates under acidic conditions.

Phenolics, particularly monophenols and diphenols, can be PPOs’ substrates or inhibitors; however, PPOs from different sources exhibits distinct substrate specificity. Previous studies have reported that certain phenolic compounds, such as ferulic acid, can inhibit PPO activity [28], while others, such as L-DOPA, tannic acid, and caffeic acid, serve as effective substrates [29,30]. Understanding PPOs’ substrate specificity is important to optimize *o*-quinone production, and hence, to improve the efficiency of PPO-mediated crosslinking. These high-specific phenolic substrates are often used as mediators when natural PPO substrates (e.g., free L-tyrosine or endogenous polyphenols) are limited, or in cases where the PPO enzyme exhibits only diphenolase activity. For example, tannic acid and caffeic acid have been shown to efficiently mediate protein crosslinking in PPO-catalyzed systems, whereas no crosslinking occurs in their absence [31,32].

This work focuses on the extraction and characterization of PPOs from apple and pear pomace and avocado seeds, with an emphasis on identifying acid-adapted PPOs suitable for low-pH food applications. Specifically, the objectives are to (1) identify PPOs with optimal and stable activity under acidic conditions, (2) characterize their properties and enzymatic activities toward various phenolic substrates, and (3) evaluate their capacity to catalyze plant protein crosslinking using phenolic mediators under acidic-pH condition.

## 2. Materials and Methods

### 2.1. Materials and Chemicals

Hass avocados (*Persea americana*), Anjou (*Pyrus communis* L.) and Bartlett (*Pyrus communis* L.) pears, Red Delicious and McIntosh apples (*Malus domestica*) were purchased at local grocery stores (State College, Pennsylvania). All chemicals, including 3,4-Dihydroxy-L-phenylalanine (L-DOPA), L-Tyrosine, β-Mercaptoethanol (β-ME), epigallocatechin gallate (EGCG), caffeic acid, ferulic acid, gallic acid, tannic acid, and soymeal enzymatic hydrolysates (a water-soluble protein hydrolysate obtained from the papainic enzyme digestion of soybean meal, listed as “peptone from soymeal” in the catalog; Cat. No. 1072120500) were purchased from Sigma-Aldrich Inc. (St. Louis, MO, USA)

### 2.2. Crude PPO Extraction

The pears and apples were processed separately using a juicer (Juilist, Shenzhen, China) operating in high-speed mode to obtain the cider (i.e., unclarified fruit juice) and pomace. The cider was directly used as cider PPO. The pomace was mixed with a sodium phosphate buffer with pH 6.5, 50 mM at a ratio of 1:3 (*w*/*v*), stirred for 1 h, and centrifuged at 11,000× *g* for 20 min to collect the supernatant that was used as pomace PPO.

The avocado seeds were obtained by manually removing the peel and flesh and washing under tap water. A high-speed electric grain mill (Cgoldenwall, Hangzhou, China) was used to grind the fresh avocado seeds into powder. This powder was mixed with a sodium phosphate buffer with pH 6.5, 50 mM at a ratio of 2:3 (*w*/*v*), stirred for 1 h, and centrifuged at 11,000× *g* for 20 min to collect the supernatant that was used as avocado seed PPO.

All extraction steps were conducted at 4 °C.

The pH of the cider was measured directly using the juice, whereas the pH of the pomace and avocado seed powder were determined by blending each sample with distilled water at a 1:1 (*w*/*v*) ratio before measurement.

### 2.3. Protein Quantification and Enzyme Activity Assay

Monophenolase was determined using a minor modification of the method from Sigma-Aldrich [33]. Briefly, a 3 mL mixture of 2900 µL of 1 mM L-tyrosine was mixed with 100 µL of an enzyme sample and incubated for 10 min (due to the lag phase), and the rate of the enzymatic reaction was determined as the increase in absorbance over the following 30 min. One unit (U) of monophenolase activity is defined as the amount of enzyme that causes an increase in absorbance at 280 nm by 0.001 per min under the mentioned conditions.

For diphenolase activity, the measurement was conducted following the method from Pretzler and co-workers [34]. Briefly, a 2 mL mixture containing 1900 µL of 1 mM L-DOPA in 50 mM sodium phosphate buffer with pH 6.5 and 100 µL enzyme sample was incubated at 25 °C. The change in absorbance was measured immediately. The initial rate of the enzymatic reaction was determined as the increase in absorbance of dopachrome (475 nm) over 3 min. One unit of diphenolase activity is defined as the amount of enzyme required to catalyze the formation of 1 µmole of dopachrome per min from L-DOPA under these conditions (1 U = 1000 nU).

Specific activity was calculated by dividing total enzyme activity (U) by the total protein content (mg). Protein was determined according to the bicinchoninic acid (BCA) assay [35] using bovine serum albumin (BSA) as a standard.

### 2.4. Optimal pH and pH Stability

The optimal pH and pH stability of the PPOs were determined following the method of Han et al., (2019) [36] with modifications. To determine the optimal pH for PPO activity, L-DOPA (1 mM) was prepared in 50 mM buffers with pH ranging from 3.0 to 8.0, citrate buffer (pH 3.0–6.2), and phosphate buffer (pH 5.8–8.0). The PPO activity assay was conducted, as previously described (Section 2.3). The pH with the highest specific activity was taken as optimal.

The pH stability was examined by incubating 100 µL enzyme samples for 12 h at 4 °C in buffer (900 µL with pH 3.0–8.0; the types of buffers at each pH were those described above). Aliquots of the enzyme sample (100 µL) were then used to determine enzyme activity and enzyme specific activity at 10 mM L-DOPA. The residual activity (%) was calculated by taking the PPO initial activity under each pH condition at 0 h as 100%.

### 2.5. Optimal Temperature and Thermal Stability

The effect of temperature on PPO activity was measured using a method described by Kolcuoǧlu (2012) [37], with modifications. L-DOPA solution (1 mM, pH 6.5) was incubated for 20 min to reach the desired temperature at 4–70 °C before adding the enzyme sample (100 µL). The enzyme activity was determined using the methods described above (Section 2.3). The temperature with the highest specific activity was considered to be optimal.

The PPO thermal stability was measured by incubating the aliquots of the enzyme sample (100 µL) at 4–70 °C for 12 h, then rapidly cooling under tap water to 25 °C. Enzyme activity was determined using the methods described above (Section 2.3). The residual activity (%) was calculated by taking the PPO initial activity at 21 °C, with pH 6.5 for 0 h at 100%.

### 2.6. Electrophoresis Study

SDS-PAGE was performed at room temperature on a BIO RAD Mini-PROTEAN Tetra system following the Laemmli [38] method, under specific conditions (with 2-mercaptoethanol) [39]. Samples were mixed with 2× Laemmli buffer (final 1×, with 50 mM DTT) and heated at 95 °C for 5 min. Protein was then loaded onto 5% stacking and 12% resolving gels and separated in Tris–Glycine–SDS running buffer (25 mM Tris, 192 mM glycine, 0.1% SDS, pH 8.3) at 80 V through the stacking and 120 V for resolving. The gels were stained with Coomassie Brilliant Blue R-250 and destained in a methanol/acetic acid solution. Precision Plus Protein All Blue Standards (#1610373, Bio-Rad) were used as molecular weight (MW) markers.

### 2.7. Substrate Specificity and Kinetic Assay

Substrate specificity was conducted according to Selinheimo et al. [29] with minor modifications. Solutions of various substrates (gallic acid, tannic acid, caffeic acid, ferulic acid, L-DOPA, and EGCG) were prepared in a citric acid buffer 50 mM with pH 5.0. A mixture of 100 µL enzyme sample and 1900 µL substrate solution with a final concentration of 5 mM was incubated at 25 °C. The absorbance spectra wavelength (350 nm–800 nm) were immediately measured at intervals over 30 min to monitor the formation of colored pigments produced by polyphenol oxidation catalyzed by the PPOs [29].

The selected substrates, along with their characteristic wavelengths identified in the above experiment, were used for a kinetic assay with a concentration range of 0.2–10 mM. The data obtained were plotted using the Lineweaver–Burk plot, and the Michaelis–Menten equation was used to determine K_m_ (the Michaelis constant) and V_max_ (the maximum reaction rate).

### 2.8. Soymeal Enzymatic Hydrolysates Crosslinking

The effect of extracted PPOs on plant protein crosslinking was determined under acid condition using EGCG and L-DOPA as mediators.

A 10% (*w*/*v*) soymeal enzymatic hydrolysates solution was prepared by dissolving soymeal enzymatic hydrolysates in citric acid buffer 50 mM, with pH 4.0 at room temperature. Aliquots of the 2 mL reaction mixtures were prepared containing 10% (*w*/*v*) soymeal enzymatic hydrolysates with 14 nU Anjou pear pomace PPO or Bartlett pear pomace PPO and 5 mM EGCG or L-DOPA. The mixtures were shake-incubated overnight at 20 °C. Reactions were stopped by mixing samples with SDS-PAGE loading buffer containing β-ME, then boiled for 10 min to terminate enzymatic reactions.

Control reactions included PPOs alone, heat-inactivated PPOs, soymeal enzymatic hydrolysates alone, and combinations of PPOs or heat-inactivated PPOs with soymeal enzymatic hydrolysates and either EGCG or L-DOPA. Heat-inactivation PPOs were prepared by incubating the PPOs at 80 °C for 20 min, followed by cooling to room temperature before use.

### 2.9. Statistical Analysis

All experiments were performed in triplicate. Statistical analyses were conducted using Minitab 20 (State College, PA, USA). Descriptive statistics, including mean and standard deviation, were calculated for each group. One-way ANOVA was used to assess significant differences among group means. If ANOVA indicated significance (*p* < 0.05), Tukey’s HSD test was performed for pairwise comparisons at a 95% confidence level.

## 3. Results and Discussion

### 3.1. Effect of pH on Activity of Polyphenol Oxidase from Agricultural Waste

Both monophenolase and diphenolase activity were investigated for all the samples before evaluating the effect of pH on each activity. L-tyrosine was used to assess the monophenolase activity; however, no increase in absorbance (280 nm) was observed in any sample over 60 min, indicating that none of the PPO extracts converted L-tyrosine to L-DOPA. This suggests that the PPOs studied lack monophenolase activity and cannot be classified as tyrosinases or laccase. Therefore, only the diphenolase activity of these fruit sources was measured in the following work.

**Figure 1:**
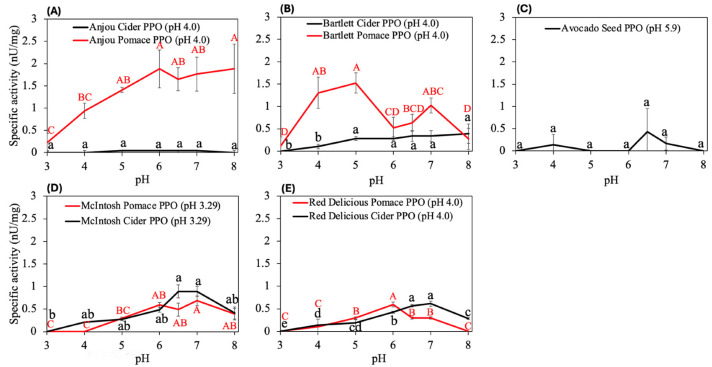
Effect of pH on PPO activity from Anjou (**A**) or Bartlett (**B**) pears, Hass avocados (**C**), McIntosh (**D**) and Red Delicious (**E**) apples. Different superscript letters are significantly different within each sample group (*p* < 0.05). Optimal pH was determined as the pH of the samples labelled A (or a). The number in parentheses is the pH of the sample.

The effect of pH (3.0–8.0) on the diphenolase activity of the PPOs from the selected agricultural by-products is shown in Figure 1. Most samples exhibited enzymatic activity across a pH range of 4.0–8.0, with optimal activity generally observed under neutral conditions (pH 6.0–8.0), regardless of the fruit’s natural acidity. Notably, Bartlett pear pomace PPO displayed the most acid-adapted profile, with peak activity observed between pH 4.0 and 5.0. This was followed by PPO Anjou pear PPO cider and pomace, with an optimal pH between 5.0 and 8.0. Previous studies have reported pH optima for PPO from different pear cultivars, including pH 4.5–5.5 in Chinese, Bosc, Anjou, and Bartlett pears [40,41,42] and pH 7.0 in Durondeau, Rocha, and Anjou pears [6,12,43]. These findings indicate that certain pear varieties possess PPOs that function better in acidic environments. For apples, the PPO’s optimal pH has generally been shown from acid to neutral pH: Red Delicious at pH 5–5.5, Granny Smith at pH 4.5 or 7.0, Golden Delicious at pH 5.5–8.0, Red Fuji at pH 8.0 [6,36,44,45,46,47]. In this study, Bartlett and Anjou pear pomace PPOs showed the most acid-adapted profiles (pH 4.0–5.0) and the highest specific activity, which were selected for further enzymatic characterization.

PPOs extracted from avocado seed showed low specific activity across all tested pH values. This is despite the rapid formation of orange pigment upon exposure of cut seeds to air, which suggests the presence of PPOs [48]. The low specific activity seen here can be due to (i) low PPO yield per extracted protein mass (35.64 mg/mL), (ii) low activity toward L-DOPA, (iii) low concentration of PPOs in avocado seed, or (iv) the pigments produced from avocado seeds that might inhibit PPO activity as reported by Dabas et al. [48]. While there are no reports on the properties of avocado seed PPOs, the PPOs from Hass avocado pulp was reported to exhibit optimal activity at values from pH 5.0 [14] to pH 7.0–7.5 [6,49].

Protein concentration in the apple and pear ciders (48.44–110.85 mg/mL) were higher than that in the pomace (25.97–35.96 mg/mL) (Table S1). However, PPOs from pear pomaces exhibited 15- to 20-fold higher specific PPO activity and more acidic optimal pH than the cider samples of the same variety at their respective optimal pH values. Previous studies have found two major types of PPOs in fruits that have different properties and specific activity: membrane-bound PPO (mPPO), which is associated with the thylakoid membrane, and soluble PPO (sPPO), which resides in the chloroplast lumen [50]. In most fruits, mPPO was shown to be more resistant to heat and low pH compared to sPPO [51,52]. However, the difference in specific activity can be related to different fruit types and varieties [51,53,54] or the extraction methods. To be specific, the supernatant from the fruit slurry can be used as sPPO, but the pellets are usually treated with temperature-induced phase partitioning method, Triton X-114, acid-shock, or anionic detergents chemistries during extraction, which may lead to 19- to 90-fold higher specific activity compared to sPPO [55,56].

**Figure 2:**
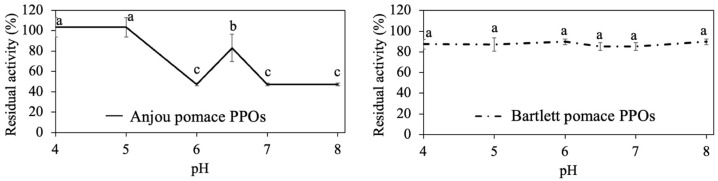
Effect of pH on stability of Anjou and Bartlett pear pomace polyphenol oxidase after 12 h; 100% of residual activity was taken as the original activity of each pH at time zero. Different superscript letters are significantly different within each sample group (*p* < 0.05).

To assess their pH stability, pomace PPOs from Bartlett and Anjou pears were incubated at various pH values (4.0–8.0) for 12 h, and the residual activity was measured (Figure 2).

Anjou pear pomace PPO retained full activity under acidic conditions (pH 4.0–5.0) but lost approximately 50% activity at alkaline pH (6.0–8.0). This result aligns with earlier findings that Anjou pear pomace PPO remained stable at 4 °C and pH 5.0 over two days [57]. In contrast, Bartlett pear pomace PPO maintained activity across the entire pH range, keeping 85% residual activity after 12 h.

### 3.2. Optimal Temperature and Thermal Stability of the Acid-Adapted Polyphenol Oxidase

**Figure 3:**
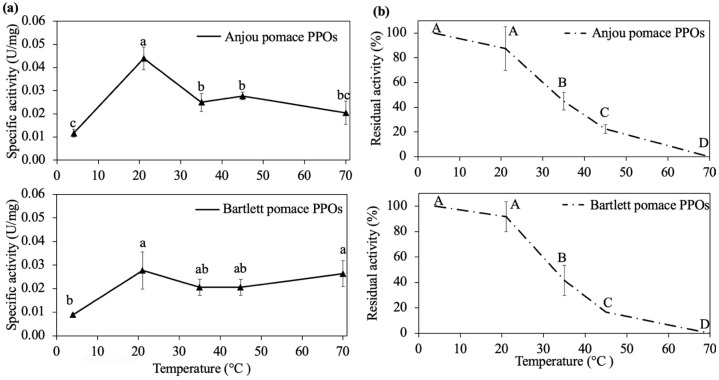
Effect of temperature on Anjou and Bartlett pear pomace polyphenol oxidase. (**a**) Optimal temperature and (**b**) thermal stability after 12 h. Residual activity was calculated based on the residual enzyme activity after thermal treatment compared to the original activity (21 °C at time 0). Different superscript letters are significantly different within each sample group (*p* < 0.05). Optimal temperature was determined as the pH of the samples labelled A (or a).

The effect of temperature (4–70 °C) on PPO activity extracted from Anjou and Bartlett pear pomace is shown in Figure 3a. Anjou pear pomace PPO showed a sharp optimal activity peak at 20 °C, while Bartlett pear pomace PPO exhibited a broader temperature tolerance, maintaining high activity from 20 °C to 70 °C. Earlier studies showed optimal temperatures around 20–23 °C for PPO from Bosc, Red, and Bartlett pears [42]. According to the literature, pears tend to be stored at a temperature of 21 °C or lower [58], which correlates with the optimal temperature found in pear PPO.

Thermal stability tests further revealed that both Anjou and Bartlett pear pomace PPOs retained activity at low to moderate temperatures (4–20 °C) but were fully inactivated after 12 h of incubation at 70 °C (Figure 3b). These results are consistent with earlier findings by Halim and Montgomery [57], who reported that Anjou PPO remained active at 4 °C for up to two days. Based on these observations, 20 °C appears to be an ideal temperature for enzyme application, while heating to 70 °C or above may serve as an effective method for PPO inactivation in processing contexts.

### 3.3. Protein Profile of the Acid-Adapted Polyphenol Oxidase

**Figure 4:**
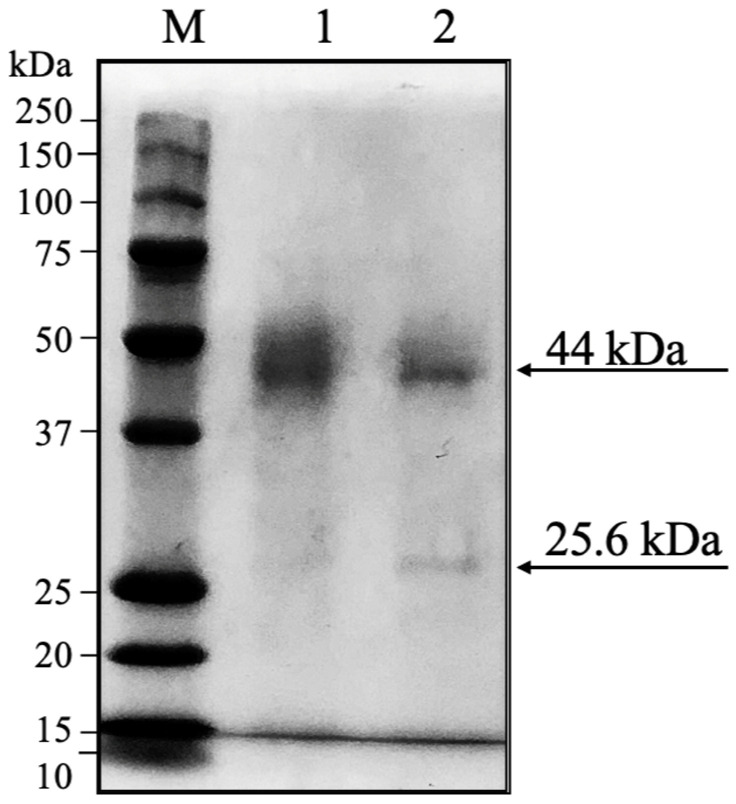
SDS-PAGE gel for protein marker (M), Anjou (Lane 1) and Bartlett (Lane 2) pear pomace polyphenol oxidase extracts. Each well contains an equal amount of 20 µg protein.

The protein profiles of crude extracts from Anjou and Bartlett pear pomace were analyzed using SDS-PAGE under reducing conditions (Figure 4). The Anjou pear pomace and the Bartlett pear pomace extract displayed two distinct bands, one at ~44 kDa and a second at ~25.6 kDa. The 44 kDa band is consistent with the molecular weight previously reported for PPOs from Bartlett pear pulp (43 kDa) [59], and similar values have been reported for PPOs from other pear varieties. For example, Whangkeumbae pear PPO was found to have a molecular weight of 44 kDa [60] and Ankara pear (*Pyrus communis*) had 3 PPO isoenzymes with MWs of 28, 40, and 60 kDa [61]. Sequence data show that *Pyrus communis* pears have multiple PPO isoforms, including a polyphenol oxidase latent form, chloroplastic-like (XP_068323921.1), with a predicted molecular weight of approximately 25.2 kDa, closely matching the 25.6 kDa band detected in the two-pear pomace PPOs (Table S2). However, SDS-PAGE provides molecular weight estimates and cannot confirm enzymatic identity. Future work should involve activity staining or purification to identify the PPO isoforms present in pear pomace.

### 3.4. Substrate Specificity and Kinetic Assay of the Acid-Adapted Polyphenol Oxidase

**Figure 5:**
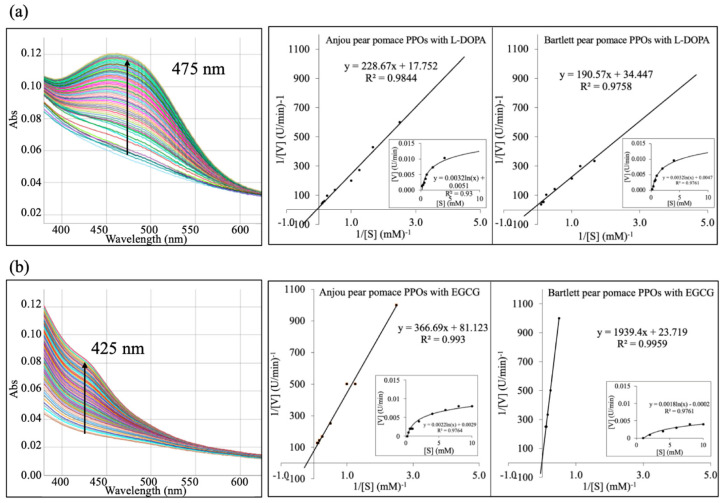
Spectral scanning over 30 min and kinetic analyses of Anjou and Bartlett pear pomace polyphenol oxidase activity using (**a**) L-DOPA and (**b**) EGCG as substrates. Absorbance spectra were recorded to monitor product formation. Michaelis–Menten and Lineweaver–Burk plots were generated based on initial reaction rates.

Given their high specific activity under acidic conditions (Section 3.1), PPOs from Anjou and Bartlett pear pomace were selected for further kinetic studies with various phenolic substrates. Among the six phenolic compounds tested, only L-DOPA and EGCG demonstrated significant enzymatic oxidation, as shown by a progressive increase in absorbance at specific visible wavelengths (Figure 5).

Spectral scanning confirmed product formation from the oxidation of L-DOPA (475 nm) and EGCG (425 nm) by both Anjou and Bartlett pear pomace PPO (Figure 5). Michaelis–Menten and Lineweaver–Burk plots were constructed, and kinetic parameters were calculated (Table 1). Both PPO oxidized L-DOPA efficiently, with Bartlett pear pomace PPO exhibiting stronger substrate affinity (K_m_ = 5.5 mM) and catalytic efficiency (V_max_/K_m_ = 0.005) compared to Anjou pear pomace PPO (K_m_ = 12.5 mM and V_max_/K_m_ = 0.004). Conversely, Anjou pear pomace PPO showed higher affinity (K_m_ = 4.2 mM) and catalytic efficiency (V_max_/K_m_ = 0.003) for EGCG, making them more suitable for EGCG-based applications. This study is the first to report detailed kinetic parameters for EGCG oxidation by pear pomace PPO. It also highlights the importance of selecting PPO sources and corresponding phenol substrates for food applications.

Gallic acid, caffeic acid, tannic acid, and ferulic acid did not exhibit any change in absorbance (Figure S1), indicating they were not oxidized under the tested conditions. This could reflect poor substrate suitability or inhibitory effects. Ferulic acid, a known PPO inhibitor, chelates copper at the enzyme’s active site and has previously been shown to inhibit pear purée PPO activity by ~90% at 15 mM [28]. Although gallic and caffeic acids have been previously identified as poor PPO substrates at higher concentrations (300 mM for gallic acid; 15–30 mM for caffeic acid) [41,43], the concentration used in this study (10 mM) may have been too low to observe significant activity.

### 3.5. Crosslinking of Soymeal Enzymatic Hydrolysates with Acid-Adapted Polyphenol Oxidase

**Figure 6:**
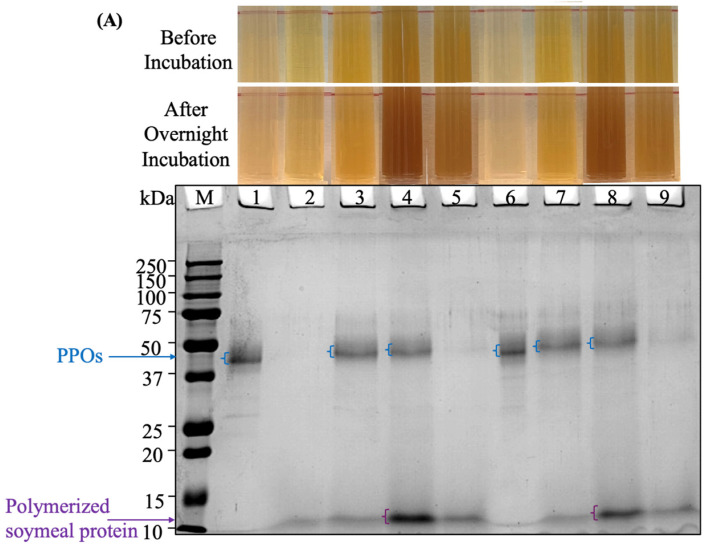

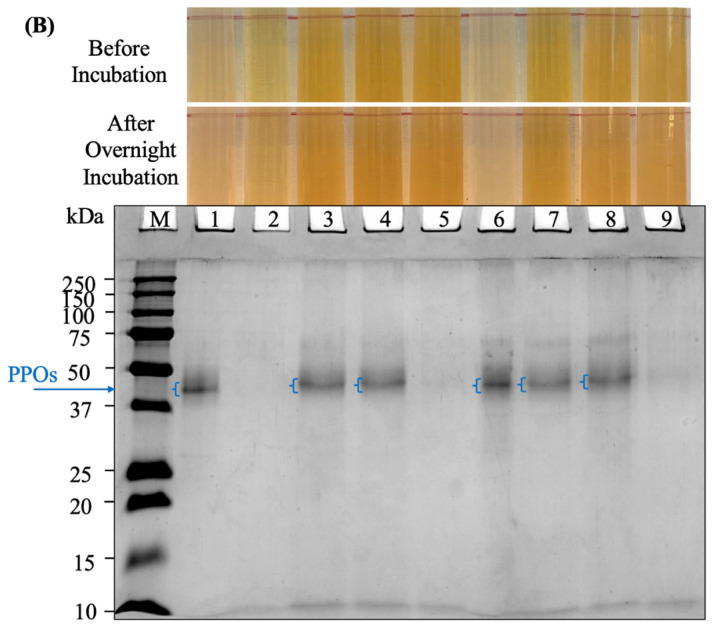
SDS-PAGE analysis of soymeal enzymatic hydrolysates crosslinking mediated by acid-adapted polyphenol oxidase at pH 4.0 using phenolic mediator (**A**) EGCG or (**B**) L-DOPA. Lane information (and amount of loaded protein in each well): Lane 1: Control-14 nU Anjour pear PPO (34.9 µg); Lane 2: Control-10% soymeal enzymatic hydrolysates (8.7 µg); Lane 3: Treatment-14 nU Anjour pear PPO + 10% soymeal enzymatic hydrolysates (43.6 µg); Lane 4: Treatment-14 nU Anjour pear PPO + 10% soymeal enzymatic hydrolysates + 5 mM L-DOPA/EGCG (43.6 µg); Lane 5: Control-14 nU inactive Anjour pear PPO + 10% soymeal enzymatic hydrolysates + 5 mM L-DOPA/EGCG (control) (43.6 µg); Lane 6: Control-14 nU Bartlett pear PPO (36.7 µg); Lane 7: Treatment-14 nU Bartlett pear PPO + 10% soymeal enzymatic hydrolysates (45.4 µg); Lane 8: Treatment-14 nU Bartlett pear PPO + 10% soymeal enzymatic hydrolysates + 5 mM L-DOPA/EGCG (45.4 µg); Lane 9: Control-14 nU inactive Bartlett pear PPO + 10% soymeal enzymatic hydrolysates + 5 mM L-DOPA/EGCG (45.4 µg). As shown in (**A**), samples containing EGCG exhibited obvious browning and cloudiness after incubation, in contrast to the control groups. This visual change indicated both EGCG oxidation to a browning pigment and the formation of large protein aggregates capable of scattering light. To confirm protein crosslinking, samples were analyzed by SDS-PAGE. Lane 4 showed a distinct band near 10 kDa, confirming that EGCG, due to its multiple phenolic groups, acts as an effective mediator for PPO-catalyzed protein crosslinking. This result demonstrates the formation of higher molecular weight aggregates from low molecular weight soy peptides under acidic conditions. In contrast, (**B**) shows that samples treated with L-DOPA exhibited no visible browning, turbidity, or high molecular weight protein bands, indicating no detectable crosslinking, although dopachrome formation (reddish color) was observed during diphenolase activity assays.

To evaluate the ability of acid-adapted PPOs to crosslink plant proteins under acidic conditions, experimental conditions were designed based on the previously determined optimal pH, temperature, and substrate specificity. The PPOs were incubated overnight with soymeal enzymatic hydrolysates in the presence or absence of 5 mM phenolic mediators (L-DOPA or EGCG). The results of the protein crosslinking experiments are presented in Figure 6. The soymeal hydrolysates, due to enzymatic hydrolysis, showed low MW bands on the SDS-PAGE gel (Figure 6, Lane 2). Effective protein crosslinking was expected to generate higher molecular weight oligomers of these peptides which would appear as distinct bands on the gel.

However, on Lanes 3 and 7, soymeal enzymatic hydrolysates treated with PPOs alone showed no change in appearance or formation of high molecular weight bands indicating there was no crosslinking. This result indicates that, in the absence of monophenolase activity, PPOs cannot crosslink pure protein by oxidizing L-tyrosine and may require di-phenolic mediators. This observation is also in agreement with previous studies where apple PPOs failed to crosslink casein in the absence of phenolic mediators [9]. Therefore, EGCG and L-DOPA were introduced as crosslinking mediators to facilitate the PPO-mediated protein conjugation process.

The structural differences between L-DOPA and EGCG may explain this result. L-DOPA contains a single phenolic ring and can theoretically form a maximum of two covalent bonds with peptides (Figure S2), which may be insufficient for building larger crosslinked networks detectable by SDS-PAGE under these conditions. In contrast, EGCG contains multiple phenolic rings and hydroxyl groups, enabling more extensive crosslinking with proteins, leading to the formation of higher molecular weight structures detectable by SDS-PAGE. Previous studies reported the difference in oxidation based on phenolic structure. For example, the oxidative polymerization of pyrogallol (which has three hydroxyl groups) proceeds faster than that of pyrocatechol (which has two). Specifically, via autoxidation in air, pyrogallol formed a free-standing film at the air–water interface via oxidative polymerization within 2 min, whereas pyrocatechol required approximately 2 h under similar conditions [62,63], which also highlights the effect of additional hydroxyl groups on the kinetics of crosslinking [52].

Another factor influencing crosslinking efficiency is PPO and phenolic compound concentration. Selinheimo et al. reported that 1000 nU of apple PPO successfully crosslinked casein in the presence of 2 mM L-DOPA, while 100 nU did not induce crosslinking. Similarly, Pei et al. showed that PPO-catalyzed crosslinking leading to soy protein isolate aggregation in the presence of tannic acid, and the degree of aggregation was positively correlated with tannic acid concentration [30]. In the current study, the concentration of pear pomace PPO may have been insufficient for effective L-DOPA-mediated crosslinking.

PPOs have been utilized to crosslink proteins and enhance their functional properties [64]. However, many PPOs exhibit optimal activity near neutral pH, limiting their application in acidic food matrices [2]. In this study, pear pomace PPO retained catalytic activity at pH 4.0, effectively oxidizing EGCG and catalyzing the crosslinking of soymeal enzymatic hydrolysates. These results demonstrate the potential of pear PPO as acid-adapted biocatalysts for protein modification in low-pH food systems. EGCG, when used as a phenolic mediator, supports the formation of polymerized protein structures. Previous studies have leveraged EGCG’s chemical structure to crosslink proteins like casein and polysaccharides such as chitosan, forming biofilms and enhancing material properties via tyrosinase catalysis [65,66]. The findings of this study provide a foundation for future food applications where agricultural by-products PPO and EGCG are to be incorporated into acidic protein systems to enhance functionality and potentially contribute nutritional benefits.

## 4. Conclusions

In conclusion, this study successfully identified the most acid-adapted PPOs among the agricultural by-products selected. While all samples exhibited some enzymatic activity within the acidic pH range of 3.0–6.0, PPOs extracted from Anjou and Bartlett pear pomace demonstrated the highest specific activity under acidic conditions (pH 4.0–6.0), making them the most promising sources of acid-tolerant PPOs. These enzymes also remained stable at pH 4.0–5.0 and 4 °C for over 12 h, with optimal catalytic activity observed at 20 °C. Regarding substrate specificity, both PPO extracts showed negligible oxidation activity toward gallic acid, caffeic acid, tannic acid, and ferulic acid. In contrast, they exhibited strong diphenolase activity by effectively oxidizing L-DOPA (Anjou K_m_ = 12.9 and Bartlet K_m_ = 5.5) and EGCG (Anjou K_m_ = 4.2 and Bartlet K_m_ = 81.8). Notably, under acidic conditions (pH 4.0), both PPOs catalyzed the crosslinking of soymeal enzymatic hydrolysates in the presence of EGCG, resulting in the formation of prominent 10 kDa bands on SDS-PAGE. These findings suggest that pear pomace PPOs are a viable biocatalyst for modifying plant proteins in acidic environments. Future research can further explore cost effective, techno-functional properties and sensory impacts, and direct application of the PPO in food systems, particularly for improving emulsion stability and viscosity.

## Figures and Tables

**Table 1:** Kinetic parameters of Anjou and Bartlett pear pomace PPO with L-DOPA and EGCG.

Substrate	Source	Wavelength (nm)	V_max_ (U/min)	K_m_ (mM)	Catalytic Efficiency (V_max_/K_m_)
L-DOPA	Anjou	475	0.056	12.9	0.004
	Bartlett	475	0.029	5.5	0.005
EGCG	Anjou	425	0.012	4.2	0.003
	Bartlett	425	0.042	81.8	0.0005

## Data Availability

The original contributions presented in this study are included in the article/Supplementary Materials. Further inquiries can be directed to the corresponding author.

## Supplementary Materials

The following supporting information can be downloaded at: https://www.mdpi.com/article/10.3390/foods14193312/s1, Figure S1. UV/VIS spectral analysis of phenolic oxidation every 5 min over 30 min by pears pomace PPO: (a) Anjou pear pomace PPO with 5 mM phenolic compounds, (b) Bartlett pear pomace PPO with 5 mM phenolic compounds (c) Anjou/Bartlett pear pomace PPO with 10 mM caffeic acid; Figure S2. Protein crosslinking mechanism of tyrosinase via (a) tyrosine amino acid function group, (b) Phenolic compound.; Table S1. Protein content in selected fruit sample extractions; Table S2. Pyrus communis pear PPO protein data obtained from NCBI.

## Abbreviations

The following abbreviations are used in this manuscript:

PPO	Polyphenol oxidase
L-DOPA	3,4-Dihydroxy-L-phenylalanine
EGCG	Epigallocatechin gallate
SDS-PAGE	Sodium dodecyl sulfate–polyacrylamide gel electrophoresis
UV-VIS	Ultraviolet–visible spectrophotometry
β-ME	β-Mercaptoethanol
U	Unit
nU	Nanounit
kDa	Kilodalton
