# Supplementary material for: Acid-Adapted Polyphenol Oxidases from Agricultural Wastes: Extraction, Characterization, and Application in Plant Protein Crosslinking
Source: Foods. 2025 Sep 24;14(19):3312. doi: 10.3390/foods14193312 (PMC12523565; doi:10.3390/foods14193312)
Supplement: Supplementary file 1 [file foods-14-03312-s001.zip › foods-3849750-supplementary.pdf]

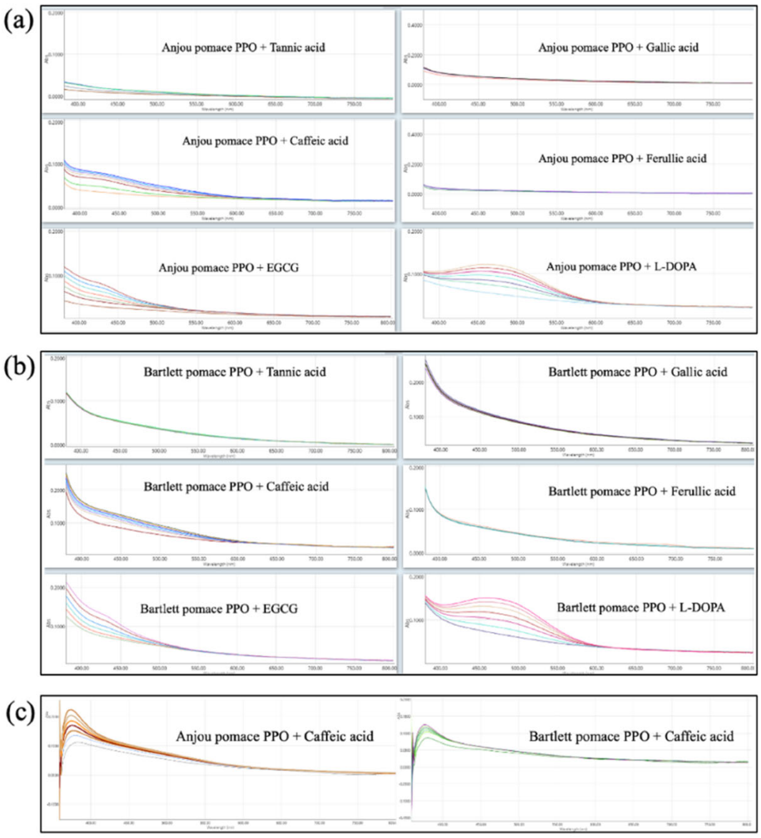

Figure **S1.** UV/VIS spectral analysis of phenolic oxidation every 5 min over 30 min by pears pomace PPO: **(a)** Anjou pear pomace PPO with 5 mM phenolic compounds, **(b)** Bartlett pear pomace PPO with 5 mM phenolic compounds **(c)** Anjou/Bartlett pear pomace PPO with 10 mM caffeic acid.

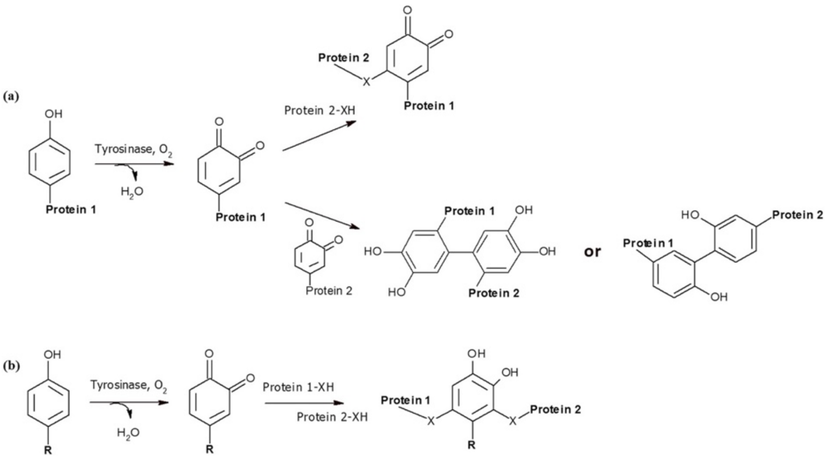

Figure **S2**. Protein crosslinking mechanism of tyrosinase via (**a)** tyrosine amino acid function group, (**b)** Phenolic compound. Adapted from Isaschar-Ovdat & Fishman, 2018 [64].

Table S1. Protein content in selected fruit sample extractions

Fruit samples	Protein concentration (mg/mL)
McIntosh apple pomace	33.75
McIntosh apple juice	48.44
Red delicious apple pomace	33.75
Red delicious apple juice	70.53
Bartlett pears juice	110.85
Bartlett pears pomace	25.97
Anjou pears juice	67.07
Anjou pears Pomace	28.41
Avocado seed pomace	35.64

Table S2. *Pyrus communis* pear PPO protein data obtained from NCBI.

Name on NCBI	Accession	Amino acid	Equivalent MW
polyphenol oxidase, chloroplastic [*Pyrus communis*]	XP_068322741.1	593 aa	65.8 kDa
polyphenol oxidase, chloroplastic-like [*Pyrus communis*]	XP_068324654.1	605 aa	67.2 kDa
**polyphenol oxidase latent form, chloroplastic-like [*Pyrus communis*]**	**XP_068323921.1**	**230 aa**	**25.52 kDa**
polyphenol oxidase, chloroplastic-like [*Pyrus communis*]	XP_068323686.1	611 aa	67.8 kDa
polyphenol oxidase latent form, chloroplastic-like [*Pyrus communis*]	XP_068323482.1	604 aa	67.0 kDa
polyphenol oxidase, chloroplastic-like [*Pyrus communis*]	XP_068323416.1	124 aa	13.76 kDa
polyphenol oxidase latent form, chloroplastic-like [*Pyrus communis*]	XP_068323415.1	610 aa	67.7 kDa
polyphenol oxidase latent form, chloroplastic-like [*Pyrus communis*]	XP_068322113.1	587 aa	65.2 kDa
polyphenol oxidase latent form, chloroplastic-like [*Pyrus communis*]	XP_068322112.1	586 aa	65.0 kDa

**polyphenol oxidase, chloroplastic-like [*Pyrus communis*]**	**XP_068320824.1**	**222 aa**	**24.6 kDa**
polyphenol oxidase latent form, chloroplastic-like [*Pyrus communis*]	XP_068320823.1	600 aa	66.6 kDa
